# Evaluating the value of Amphiregulin, Phosphatase and Tensin Homologue *(PTEN)* and *P21* Expression for Anti-EGFR Treatment in Metastatic Colorectal Carcinoma

**DOI:** 10.31557/APJCP.2021.22.4.1025

**Published:** 2021-04

**Authors:** Ahmed Ali Obaya, Amrallah A Mohammed, Hanaa Rashied, Adel Mahmoud Morsy, Gamal Osman, Ahmed S Allam, Ahmed M Elsayed, Ola A Harb, Walid S H

**Affiliations:** 1 *Department of Clinical Oncology & Nuclear Medicine, Faculty of Medicine, Zagazig University, Egypt. *; 2 *Department of Medical Oncology, Faculty of Medicine, Zagazig University, Egypt. *; 3 *Department of Clinical Oncology & Nuclear Medicine, Elmabara Hospital of Zagazig, Health Insurance Organization, Zagazig, Egypt. *; 4 *Department of General Surgery, Faculty of Medicine, Zagazig University, Egypt. *; 5 *Department of Internal Medicine, Faculty of Medicine, Zagazig University, Egypt. *; 6 *Department of Tropical Medicine, Faculty of Medicine, Zagazig University, Egypt. *; 7 *Department of Pathology, Faculty of Medicine, Zagazig University, Egypt. *

**Keywords:** Anti-EGFR treatment, metastatic colorectal carcinoma, amphiregulin, PTEN, P21-immunohistochemistry

## Abstract

**Background:**

Despite the significant progress in target therapy for the treatment of metastatic colorectal carcinoma (mCRC), the overall survival isn’t satisfactory.

**Methods:**

We assessed the expression of *Amphiregulin, PTEN*, and *P21* in sections from 23 paraffin blocks prepared from 23 patients with left-sided mCRC using immunohistochemistry (IHC). The relationship between their level of expressions, clinicopathological parameters, response to anti-EGFR, and prognosis were analyzed.

**Results:**

High *Amphiregulin, PTEN* and low* P21* expression levels were associated with low tumor grade (p= 0.038 and 0.025 respectively), better response to anti-EGFR treatment (p <0.001), and favorable outcome {progression-free survival (PFS) and overall survival (OS)} (p <0.05). There was a direct relation between *Amphiregulin *and *PTEN* expressions (phi coefficient=+0.840), while there was an inverse relation between *P21*expression and both *Amphiregulin* (phi coefficient= -0.840) and *PTEN* expressions (phi coefficient = -1.000), which was statistically significant (P <0.001).

**Conclusion:**

High *Amphiregulin* and *PTEN *expression levels and low *P21* expression levels were associated with better response to anti-EGFR therapy and improved survival outcome. They might be considered predictive markers of response to anti-EGFR therapy in mCRC.

## Introduction

Colorectal carcinoma (CRC) is the fourth commonest diagnosed cancer and the second cause of cancer-related mortality in the United States (Siegel et al., 2019). Although the advances in the pathophysiology understanding of CRC, still its management is challenging and incurable. Anti-epidermal growth factor receptor (EGFR) combined with chemotherapy is now the standard of care in left side mCRC with *EGFR* sensitizing mutation (Khelwatty et al., 2013). The median overall survival (OS) improved 4.5 months by the addition of anti-EGFR monoclonal antibody (cetuximab) to chemotherapy (CT) protocol (Douillard et al., 2010).

However, it was found that certain patients have acquired a certain degree of drug resistance which limits its clinical efficacy (Khelwatty et al., 2017). Some studies showed that there are several mutations that occurred in many downstream effector molecules of the EGFR signaling pathway indicating that additional markers are needed to predict the response to anti-EGFR therapy (Chen et al., 2015).

Amphiregulin is one of the ligands of the EGF family that mediates the biological roles through binding to EGFR in both epithelial and mesenchymal cells (Yarden et al., 2001). Thus, Amphiregulin participates in cancer cell proliferation, migration, invasion, and angiogenesis in human carcinomas (Ma et al., 2001), so Amphiregulin was incriminated as a predictive biomarker of anti-EGFR therapy for most EGFR-driven carcinomas (Zarkavelis et al., 2017). 

Phosphatase and tensin homologue (*PTEN*) is an important cancer-suppressor gene and one of EGFR downstream cascade members. Loss of PTEN function has been reported in mCRC (Negri et al., 2010).

P21 and P27 are cyclin-dependent kinase (CDK) inhibitors linked with an increase in the cell number G2/M phase of the cell cycle. Anti-EGFR could induce cell cycle arrest at G2/M and at the G1 phase (Terzuoli et al., 2017). 

Although there are several studies that assessed predictive markers of cetuximab therapy in patients with mCRC there is no previous study that clarified the role of Amphiregulin, *PTEN*, and *P21 *expression together.

In the present study, we aimed to assess Amphiregulin, *PTEN* and *P21* expression expression in mCRC patients to correlate their levels of expression with clinicopathological criteria of the tumor and with the outcome of cetuximab sensitivity treatment. 

## Materials and Methods

This is a prospective study involving all patients with left side CRC who were operated at General Surgery Department and treated in Medical Oncology and Clinical Departments, Zagazig University hospitals during the period from December 2016 to December 2018. Patients who were clinically suspected to have CRC underwent full clinical examination, radiological evaluation in the form of pelvic abdominal computed tomography, and colonoscopy biopsies were taken and histopathological confirmation in Pathology Department, Faculty of Medicine, Zagazig University. All patients had received the specific post-operative protocol.

The inclusion criteria were age ≥18 years old, pathological diagnosed CRC, left-sided wild RAS type with a metastatic disease either denovo or after adjuvant therapy. they started the systemic treatment with a cetuximab-based protocol. Only 23 patients were eligible. The samples were prepared, diagnosed, graded, and staged according to the eighth edition of the American Joint Committee on Cancer staging system (AJCC-8) classification and the World Health Organization (WHO) classification (Amin et al., 2017, Ueno et al., 2012). Clinical and pathological criteria have been identified by review of the patient’s files. Patients were followed for 2 years from the date of metastatic disease diagnosis.

*Immunohistochemical staining*

IHC was performed on the 23 samples as previously explained (Hsu et al., 1981). The sections were incubated with the primary goat polyclonal antibody to Amphiregulin (Millipore, Billerica, MA, USA), primary rabbit monoclonal antibody to PTEN (Millipore, Billerica, MA, USA), primary mouse monoclonal antibody to p21 [EPR362] (ab109520) (Abcam, UK) dilution 1;200 for 30 min, at room temperature. The bound primary antibodies were detected by adding anti-goat secondary antibodies for 30 min, at room temperature. The sections were counterstained with Mayer’s hematoxylin, and were finally mounted.

The expression of *Amphiregulin* was found in the cytoplasm and the membrane of tumor cells, the expression of *PTEN* was in the cytoplasm and nucleus of tumor cells, and the expression of *P21 *expression was in the nucleus of tumor cells. 

*Scoring criteria*

CRC tumor section immunostaining assessed and scored depending on the percentage of stained tumor cells assigning a score of 0 to 3. Score 0= stained tumor cell > 5%, score 1= stained tumor cell > 10%, score 2= stained tumor cell > 20%, and score 3= stained tumor cell > 50%. Also, the intensity of immunostaining scored from 0-3 based on the color intensity. Score 0 = negative, score 1+ = weak light brown, score 2+= moderately brown, and score 3+= strong intense brown). To reach a final stain score of 0-9 we multiplied scores of the intensity and the extent and we considered 3 as a cut point above which is considered high expression and below which a low expression (Khelwatty et al., 2017). Correlations were done between the used markers, response to Cetuximab-FOLFIRI protocol, and survival.

Before starting the work, we obtained the institutional review board (IRB) approval with no funding support. 

*Statistical analysis*

Percent of the included categorical variables in the study were compared through using appropriate tests whether; Pearson’s Chi-square test or Fisher’s exact test. The strength of resulted relationship between expressions of* Amphiregulin, PTEN,* and *P21* were assessed by calculation of the phi coefficient considering (+) sign as an indicator for the direct relationship and considering the (-) sign as an indicator for the inverse relationship. Overall Survival (OS) rate was considered as the time from CRC diagnosis to the time of patients’ death or the time of most recent follow-up time (censored). Progression-Free Survival (DFS) rate was considered as the time from starting CRC treatment to the date of its progression or to date of patients most recent follow-up time during which the patients were progression-free. Stratification and categorization of OS and PFS rates were assessed in relation to *Amphiregulin, PTEN,* and* P21* expression and were estimated by using the method of Kaplan-Meier plot, and were compared by using the two-sided exact log-rank test. A p-value <0.05 was considered significant. Statistics of the current study were done by using SPSS 22.0 for windows (SPSS Inc., USA) and MedCalc windows (MedCalc Software bvba 13, Belgium).

## Results

*Patient data*

The clinical data of the 23 patients with mCRC that were included in the study are summarized in Table 1. 

The 23 mCRC cases included into 16 (69.6%) males and 7 (30.4%) females. The 23 patients who received cetuximab were analyzed as; 2 (8.7%) patients experienced a partial response (PR) and 13 patients (56.5%) experienced a complete response (CR). So, the overall response (OAR) rate was 65.2%. Four (17.4%) patients experienced stable disease (SD) for more than eight weeks, while four (17.4%) patients experienced a short-term progression of cancer (PD). The overall rate of disease control was 68.8%. There is no significant difference between the rate of response to therapy and patients; age, sex, histopathological subtype, site, or number of distant metastases (p > 0.05). 

**Figure 1:**
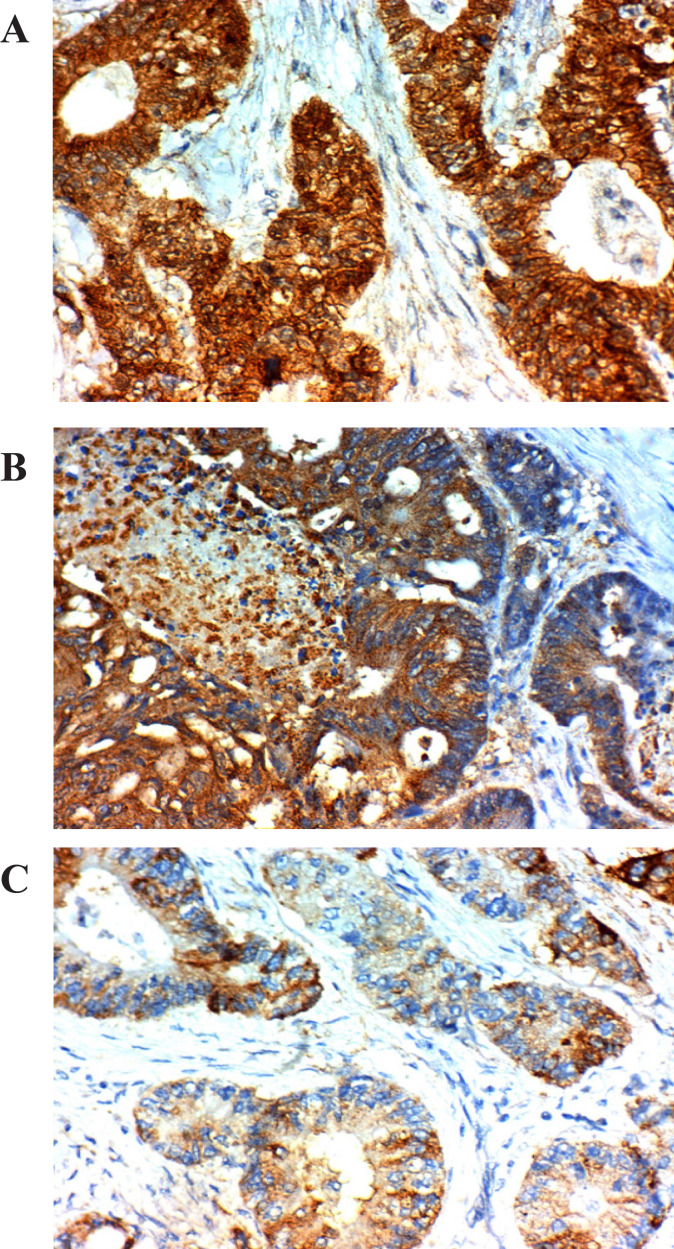
*Amphiregulin, PTEN *and *P21* Expression in Relation to Clinicopathological Parameters

**Figure 2:**
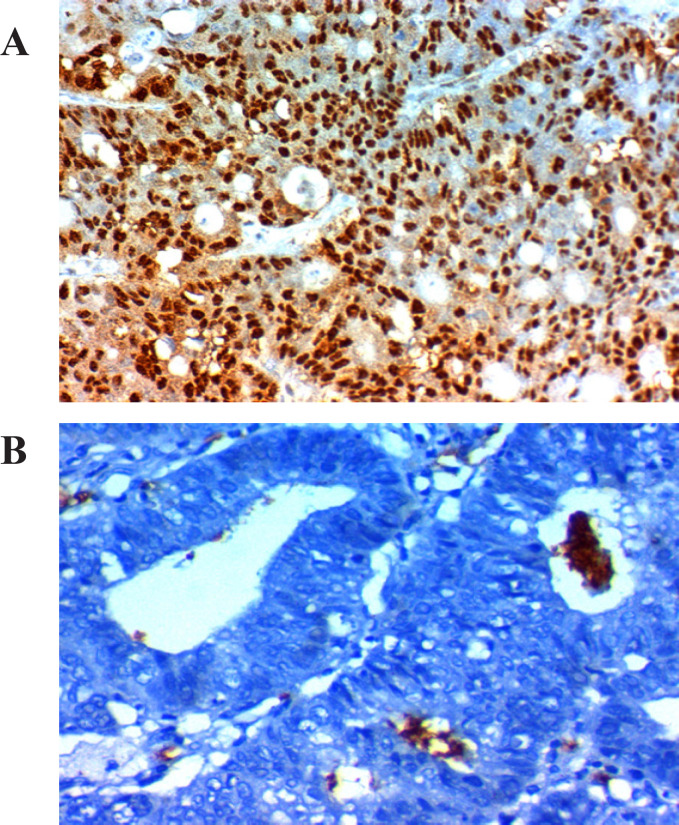
*Amphiregulin, PTEN *and *P21* Expression in Relation to Clinicopathological Parameters

**Figure 3:**
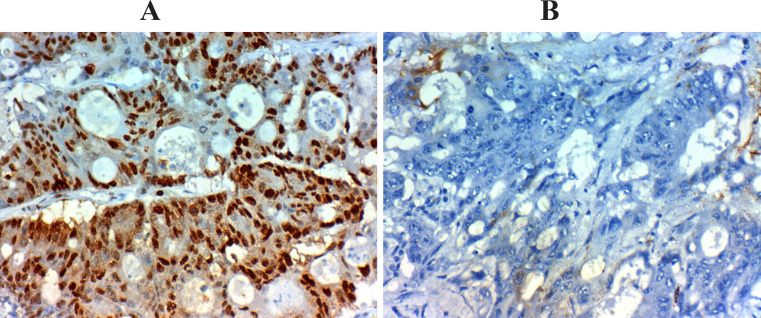
*Amphiregulin, PTEN *and *P21* Expression in Relation to Clinicopathological Parameters

*Amphiregulin, PTEN *and *P21* expression in relation to clinicopathological parameters; Table 2, Figures 1, 2 and 3

Levels of expression of *Amphiregulin, PTEN*, and *P21* were not correlated with age, sex, histopathological subtype, site, or number of distant metastases. 

High *Amphiregulin, PTEN*, and low* P21* expression levels were associated with a low grade of the tumor (p= 0.038 and 0.025 respectively) (p > 0.05).

**Figure 4:**
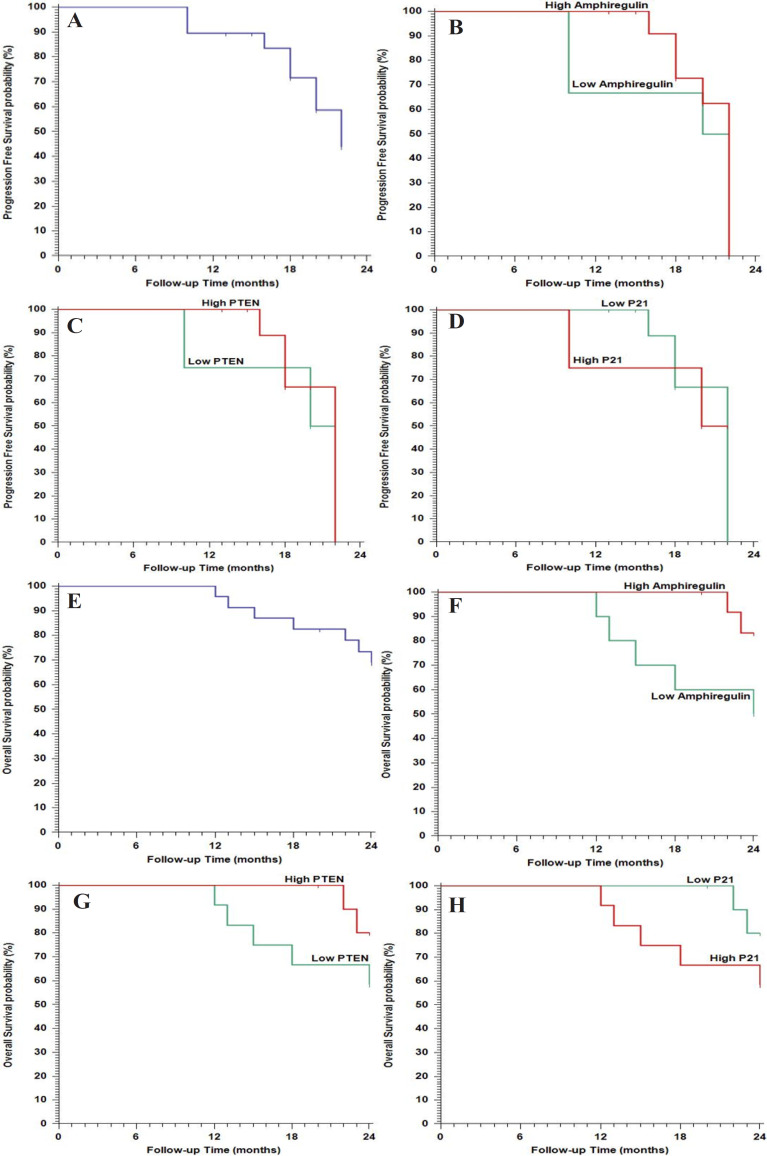
Association of *Amphiregulin, PTEN* and *P21* with Treatment Outcome

Association of *Amphiregulin, PTEN* and *P21* with treatment outcome; Table 3, Figure 4

High Amphiregulin and PTEN levels and low P21 levels were associated with cetuximab-based regimen-responsive patients (p <0.001) and better outcomes in both PFS and OS (p <0.05).

Regarding the relation between their expressions, there was a direct relation between Amphiregulin expression and *PTEN* expression (phi coefficient=+0.840), an inverse relation between Amphiregulin expression and* P21* (phi coefficient= -0.840), and an inverse relation between *PTEN* expression and *P21* expression (phi coefficient= -1.000) (P <0.001).

**Table 1:** Clinicopathological Features, Amphiregulin, *PTEN, P21* Expression and Outcome of Included Patients with Metastatic Colorectal Cancer (mCRC)

Characteristics	All patients (N=23)		Characteristics	All patients (N=23)	
	No.	%		No.	%
Age			Amphiregulin		
Mean±SD	64.69 ±7.47		Low	10	43.50%
Median (Range)	67 (50 – 75		High	13	56.50%
≤65 years	11	47.80%			
>65 years	12	52.20%			
Sex			PTEN		
Male	16	69.60%	Low	12	52.20%
Female	7	30.40%	High	11	47.80%
Histopathology			P21		
Conventional	20	87%	Low	11	47.80%
Mucinous	3	13%	High	12	52.20%
Grade			Response		
Grade I	3	13%	PD	4	17.40%
Grade II	9	39.10%	SD	4	17.40%
Grade III	11	47.80%	PR	2	8.70%
T			CR	13	56.50%
T2	5	21.70%	NR	8	34.80%
T3	7	30.40%	OAR	15	65.20%
T4	11	47.80%			
Site of metastasis			Follow-up duration (months)		
Liver only	12	52.20%	Mean±SD	22.04 ±3.78	
Liver & Other organ	11	47.80%	Median (Range)	24 (12 – 24)	
Liver metastasis			Outcome		
Single	12	52.20%	Progression	8	42.10%
Multiple	11	47.80%	Died	7	30.40%

**Table 2:** Correlations between Clinicopathological Features, *Amphiregulin, PTEN* and *P21* Expression in Included Patients with mCRC

Characteristics	All (N=23)		Amphiregulin				p-value	PTEN				p-value	P21				p-value
			Low (N=10)		High (N=13)			Low (N=12)		High (N=11)			Low (N=11)		High (N=12)		
	No.	(%)	No.	(%)	No.	(%)		No.	(%)	No.	(%)		No.	(%)	No.	(%)	
Age																	
≤65 years	11	(47.80%)	7	(63.60%)	4	(36.40%)	0.100‡	7	(63.60%)	4	(36.40%)	0.292‡	4	(36.40%)	7	(63.60%)	0.292‡
>65 years	12	(52.20%)	3	(25%)	9	(75%)		5	(41.70%)	7	(58.30%)		7	(58.30%)	5	(41.70%)	
Sex																	
Male	16	(69.60%	8	(50%)	8	(50%)	0.405‡	10	(62.50%)	6	(37.50%)	0.193‡	6	(37.50%)	10	(62.50%)	0.193‡
Female	7	(30.40%)	2	(28.60%)	5	(71.40%)		2	(28.60%)	5	(71.40%)		5	(71.40%)	2	(28.60%)	
Histopathology																	
Conventional	20	(87%)	8	(40%	12	(60%)	0.560‡	10	(50%)	10	(50%)	1.000‡	10	(50%)	10	(50%	1.000‡
Mucinous	3	(13%)	2	(66.70%)	1	(33.30%)		2	(66.70%)	1	(33.30%)		1	(33.30%)	2	(66.70%)	
Grade																	
Grade I	3	(13%)	0	(0%)	3	(100%)	0.038§	0	(0%)	3	(100%)	0.025§	3	(100%)	0	(0%)	0.025§
Grade II	9	(39.10%)	3	(33.30%	6	(66.70%)		4	(44.40%)	5	(55.60%)		5	(55.60%)	4	(44.40%)	
Grade III	11	(47.80%)	7	(63.60%	4	(36.40%)		8	(72.70%)	3	(27.30%)		3	(27.30%	8	(72.70%)	
T																	
T2	5	(21.70%)	0	0%	5	(100%)	0.001§	1	(20%)	4	(80%)	0.012§	4	(80%)	1	(20%)	0.012§
T3	7	(30.40%)	1	(14.30%)	6	(85.70%)		2	(28.60%)	5	(71.40%)		5	(71.40%)	2	(28.60%)	
T4	11	(47.80%	9	(81.80%)	2	(18.20%)		9	(81.80%)	2	(18.20%)		2	(18.20%	9	(81.80%)	
Site of metastasis																	
Liver only	12	(52.20%	3	(25%)	9	(75%)	0.100‡	4	(33.30%)	8	(66.70%)	0.059‡	8	(66.70%)	4	(33.30%)	0.059‡
Liver & Other organ	11	(47.80%)	7	(63.60%)	4	(36.40%)		8	(72.70%)	3	(27.30%)		3	(27.30%)	8	(72.70%)	
Liver metastasis																	
Single	12	(52.20%)	3	(25%)	9	(75%)	0.100‡	4	(33.30%)	8	(66.70%)	0.059‡	8	(66.70%	4	(33.30%)	0.059‡
Multiple	11	(47.80%)	7	(63.60%)	4	(36.40%)		8	(72.70%)	3	(27.30%)		3	(27.30%	8	(72.70%)	
Amphiregulin																	
Low	10	(43.50%)						10	(100%)	0	(0%)	<0.001‡	0	(0%)	10	(100%)	<0.001‡
High	13	(56.50%)						2	(15.40%)	11	(84.60%)		11	(84.60%)	2	(15.40%)	
PTEN																	
Low	12	(52.20%)	10	(83.30%)	2	(16.70%)	<0.001‡						0	(0%)	12	(100%)	<0.001‡
High	11	(47.80%)	0	(0%)	11	(100%)							11	(100%)	0	(0%)	
P21																	
Low	11	(47.80%)	0	(0%)	11	(100%)	<0.001‡	0	(0%)	11	(100%)	<0.001‡					
High	12	(52.20%)	10	(83.30%)	2	(0%)		12	(100%)	0	(0%)						

‡, Chi-square test; §, Chi-square test for trend

**Table 3:** Correlations between *Amphiregulin, PTEN* and *P21 *Expression and Outcome in Included Patients with mCRC

Characteristics	All (N=23)		Amphiregulin				p-value	PTEN				p-value	P21				p(value
			Low (N=10)		High (N=13)			Low (N=12)		High (N=11)			Low		High		
													(N=11)		(N=12)		
	No.	(%)	No.	(%)	No.	(%)		No.	(%)	No.	(%)		No.	(%)	No.	(%)	
Response																	
PD	4	(17.40%)	4	(40%)	0	0%	<0.001‡	4	(33.30%)	0	0%	0.001‡	0	0%	4	(33.30%)	0.001‡
SD	4	(17.40%)	4	(40%)	0	0%		4	(33.30%	0	0%		0	0%	4	(33.30%)	
PR	2	(8.70%)	2	(20%)	0	0%		2	(16.70%)	0	0%		0	0%	2	(16.70%)	
CR	13	(56.50%)	0	0%	13	(100%)		2	(16.70%)	11	(100%)		11	(100%	2	(16.70%)	
NR	8	(34.80%)	8	(80%)	0	0%	<0.001‡	8	(66.70%)	0	0%	0.001‡	0	0%	8	(66.70%)	0.001‡
OAR	15	(65.20%	2	(20%)	13	(100%)		4	(33.30%)	11	(100%)		11	(100%	4	(33.30%)	
Progression																	
Absent	11	(57.90%)	3	(50%)	8	(61.50%)	0.049‡	4	(50%)	7	(63.60%)	0.048‡	7	(63.60%	4	(50%)	0.0398‡
Present	8	(42.10%)	3	(50%)	5	(38.50%)		4	(50%)	4	(36.40%)		2	(36.40%	6	(50%)	
PFS																	
Mean (months)	19.64months		17.67months		20.51months		0.0486†	18.50months		20.44months		0.0494†	20.44months		18.50months		0.0384†
(95%CI)	(17.80(21.48)		(13.29(22.04)		(19.12(21.91)			(15.05(21.94)		(18.73(22.15)			(18.73(22.15)		(15.05(21.94)		
Median (months)	22 months		20 months					20 months		22 months			22 months		20 months		
12month PFS	89.50%		66.70%		100%			75%		100%			100%		75%		
18month PFS	71.60%		50%		72.70%			75%		66.70%			66.70%		75%		
Mortality																	
Alive	16	(69.60%)	5	(50%)	11	(84.60%)	0.169‡	7	(58.30%)	9	(81.80%)	0.371‡	7	(58.30%	9	(81.80%)	0.371‡
Died	7	(30.40%)	5	(50%)	2	(15.40%)		5	(41.70%)	2	(18.20%)		5	(41.70%	2	(18.20%)	
OS																	
Mean (months)	22.21months		20.20months		23.75months		0.045†	20.83months		23.70months		0.015†	23.70months		20.83months		0.025†
(95%CI)	(20.57(23.84)		(16.82(23.57)		(23.41(24.08)			(17.87(23.78)		(23.30(24.09)			(23.30(24.09)		(17.87(23.78)		
Median (months)	NR		24 months		NR			NR		NR			NR		NR		
12month OS	95.70%		90%		100%			91.70%		100%			100%		91.70%		
18month OS	82.60%		60%		100%			66.70%		100%			100%		66.70%		
24month OS	68.80%		50%		83.30%			58.30%		80%			80%		58.30%		

‡, Chi-square test; †, Log rank test

## Discussion

Despite the effectiveness of cetuximab regarding the management of patients with mCRC, it showed a considerable number of those patients appear not to benefit from it due to acquired or inherited drug resistance in some patients (Zhang et al., 2018). So, we need to identify novel biomarkers that could predict the efficacy of cetuximab therapy and allow for more effective clinical use of such drug. Molecular targeted monoclonal antibody treatment is expensive, so choosing the best biomarkers which will be adequately used to target the real patient population is a must. Additionally, choosing patients who will have a large benefit from such agents will protect them from the unneeded side effects (Sahin et al., 2014). We have chosen three biomarkers and evaluated their expression in mCRC to predict cetuximab efficacy, moreover, we used an easy and cheap technique (IHC).

In the current study, patients with high expression of *Amphiregulin* in their tumors showed good response to the cetuximab-based regimen and improved both PFS and OS. This result was consistent with the finding of previous studies that demonstrated the association between high expression of *Amphiregulin* and better cetuximab efficacy (Okada et al., 2014, Ford et al., 2007). However, the methods of Amphiregulin evaluation were different; Yonesaka et al., (2015) detected the Amphiregulin level in the plasma, Jacobs et al., (2009) assessed the gene expression levels in primary tumors by grouping them according to the status of *KRAS* mutations, Sunakawa et al., (2016) used *amphiregulin mRNA* expression and Li et al., (2010), detected the Amphiregulin levels of expression in both tumor tissue and serum of patients with mCRC and found that *Amphiregulin *expression is correlated with the degree of malignant invasion, presence of distant metastasis, peri-neural and vascular invasion, which are considered unfavorable parameters for prediction of OS and PFS.

Furthermore, Ferraros et al., (2012) had proved that suppression of *EGFR* ligand *Amphiregulin* leads to cetuximab resistance which was in line with our results and explained them.

Clearly, Amphiregulin significantly affects the prognosis of mCRC patients who are treated with cetuximab-based therapy significantly; but, patients’ outcomes could not be explained by a single factor (Yonesaka et al., 2015). Previous studies found that some mCRC patients abundantly expressing *Amphiregulin *but do not respond to cetuximab (Yonesaka et al., 2015, Ferraros et al., 2012), which could be explained by the presence of different molecules that affects cetuximab therapy. So, all ligands of EGFR should be evaluated to predict its response.

Moreover, in the present study loss of PTEN protein expression in mCRC tissues was associated with poor response to cetuximab while high levels of *PTEN *expression were associated with a better response. These results matched with the finding of Zhang et al., 2018 who found that PTEN deficiency predicts cetuximab resistance in mCRC.

Also, Chen, et al., (2015) have also shown that *PTEN *expression was associated with better OS rate and OAR to chemotherapy combined with cetuximab. The same findings were proved in many previous studies (Loupakis et al., 2009, Razis et al., 2008, Frattini et al., 2007, Mao et al., 2010, and Sood et al., 2012).

The mechanism of cetuximab resistance might be based on exosome-induced PTEN deletion. Tumor-derived exosomes participate in many processes, like tumor metastasis, drug resistance, and drug delivery (Shao et al., 2016, Zhang et al., 2018).

A functional interaction was detected between PTEN activity, EGFR tyrosine kinase signaling. PTEN was found to be related to EGFR-inhibitors resistance in many types of cancer (Mellinghoff et al., 2007). Such resistance is related to the negative regulator of the phosphatidylinositol 3’ kinase (PI3K) complex. Inactivation of PTEN leads to uncontrolled signaling of the protein kinase B (PKB)/Akt pathway that in turn dissociates the inhibition of EGFR through this pathway. As wild-type PTEN is relatively common (60–100%) in CRC, so, it is more likely that PTEN is one of the important parameters which determine response to this therapeutic monoclonal antibody (Chen et al., 2015). Contrary to our results Karapetis et al., 2013, reported that in chemotherapy-refractory CRC, *PTEN* expression was neither prognostic nor predictive of benefit from cetuximab-based therapy. 

The divergence of the results may be due to different methods of assessment and evaluation between tissue microarray and within cores. In addition, Loupakis et al., (2019) observed that the predictive value of PTEN was noticed only when the evaluated tumor tissue came from a metastatic tumor but not from the primary site. 

Notably, loss of P21 is associated with better response to cetuximab based therapy while overexpression is associated with resistance to it, which were demonstrated in many previous studies (Ogino et al., 2005, Spano et al., 2008, and Jeffrey et al., 2010), which was inconsistent with our results.

Analysis of cell cycle progression in malignant cells gives a rationale for cetuximab and P21functional effects, pointing to the underlying mechanism of action (Terzuoli et al., 2017). Cetuximab has an important role in the induction of growth arrest at the G2/M phase. The increasing number of cells at G2/M phase was related to induction of the CDK inhibitors; p21 and p27 through the silence of cyclin E/CDK2 which is the G1/S-promoting kinases that lead to arrest G1 arrest. G1/S checkpoint, which is controlled by two mechanisms related to p21 and mostly deregulated in CRC cells. Reduced the expression of *cyclin B* in cetuximab treated cells, leads to an adequate blocking of the G2/M and prevents cells from initiation of mitosis (Abbas et al., 2009, Kastan et al., 2004). Also, EGF/EGFR system impairment occurred by the cetuximab was found to be related to the ability to initiate a growth arrest in phases of G2/M in CRC cells that subsequently lead to a decrease in P21 (Li X et al..2010).

In this study, we also showed that there is an inverse association between *P21* expression and *OAR* to cetuximab in mCRC, and both act in CRC cells by two related mechanisms. First, they could accelerate EGFR turnover by ubiquitination, and decrease EGFR receptor density, allowing less concentration of cetuximab to exert cancer inhibitory effects. Second, they lead to arrest in the cell cycle at the G2/M phase by increasing expression of the *CDK* inhibitors *p21* and *p27* to increase the apoptotic process (Huang et al., 2017, Coccia et al., 2016, and Kim et al., 2016).

*limitations*

The small sample size represented the main limitation. Moreover, the cause of death was not analyzed if it is therapy-related or disease-related. Further validation by using a large cohort of patients is needed.

In Conclusion, cetuximab proved efficacy in management of mCRC but it was found that not all patients respond to such therapy. Due to its high cost, it is better to identify markers that could be able to predict the response before starting therapy. As the IHC is a cheap and easy method of tissue protein markers assessment, we used such technique to detect the predictive roles of three biomarkers which are *Amphiregulin, PTEN* and P*21*. Our study revealed that high expression of both Amphiregulin, *PTEN* in addition to *P21* down regulation were associated with better response and improved outcome to cetuximab-based therapy which suggest that assessment of tissue protein expression of those marker might be beneficial in the therapy selection. 

## Author Contribution Statement

None.
